# Editorial for the Special Issue, “Quality Assay, Processing and Bio-Function of Rice Products”

**DOI:** 10.3390/foods11121755

**Published:** 2022-06-15

**Authors:** Ken’ichi Ohtsubo, Carla Moita Brites, Cristina M. Rosell

**Affiliations:** 1 Faculty of Applied Life Sciences, Niigata University of Pharmacy and Applied Life Sciences, 265-1, Higashijima, Akiha-ku, Niigata 956-8603, Japan; 2 National Institute for Agrarian and Veterinarian Research (INIAV), I.P., Av. da República, Quinta do Marquês, 2780-157 Oeiras, Portugal; carla.brites@iniav.pt; 3 Faculty of Agricultural and Food Sciences, University of Manitoba, Ellis Building, 13 Freedman Crescent, Winnipeg, MB R3T 2N2, Canada; cristina.rosell@umanitoba.ca

Rice is one of the most important cereals in the world alongside wheat and maize. It has been pointed out that more than half of world population live on rice grains, mainly consumed as table rice, but it can be processed into various products, such as bread, noodles, and cake. In addition to traditional rice products, novel rice products have been developed and introduced using various new processing technologies [1,2]. This trend has increased the consumption of rice and rice-based foods, requiring quality evaluations and also the assessment of their nutritional and health impacts.

Quality evaluations of rice are performed via sensory testing and physicochemical measurements [3,4]. The former is a basic method that requires many samples and several panelists. The latter is an indirect method that estimates the eating quality based on the chemical composition, cooking quality, gelatinization properties, and physical properties of cooked rice. Novel methods to evaluate the quality of the cooked rice are necessary to breed high-quality rice cultivars and to select the suitable rice for each consumer and each purpose.

Concerning nutrition and health, the intake of rice grains provides the intake of calories, primary functions, sensory satisfaction, secondary functions, and the maintenance and promotion of health, as tertiary functions [5]. Scientific reports on the bio-functional effects of functional substances are increasing year by year. The bioactive components of grains are very useful for maintaining a long and healthy life.

We hope that this Special Issue will be useful for all readers in terms of the novel information it provides on rice science and technology, helping us all to lead happy and healthy lives. Summaries of each paper are as follows:

(1) The granule-bound starch synthase (GBSS) enzyme controls amylose biosynthesis in rice with two isoforms: GBSSI carries amylose in endosperm and GBSSII in leaves. Research on the functional properties of GBSSII gene in rice is limited. Maung et al. [6], in the study ‘Functional Haplotypes and Evolutionary Insight into the Granule-Bound Starch Synthase II (GBSSII) Gene in Korean Rice Accessions (KRICE_CORE)’, identified several different functional alleles or haplotypes of the GBSSII gene in 475 rice accessions. In total, 38 haplotypes were detected in 13 cultivated, 21 wild, and 4 mixed (cultivated and wild) varieties. A genetic analysis revealed relationships among cultivated and wild rice groups, providing evolutionary insights into GBSSII, whose allele diversity can be used for selecting desired traits in rice-variety breeding programs.
(2) The sparkling draft cloudy sake, AWANAMA, can be treated by high hydrostatic pressure (HHP) technology to suppress its over-fermentation. It has a potentially high market value due to its fresh flavor and fruity taste compared to traditional thermal-pasteurized sake. However, further improvements to its brewing process for commercialization still require new HHP techniques at lower pressure levels and the development of pressure-sensitive (piezosensitive) sake yeast strains. Shigematsu et al. [7], in their study ‘The Genomic and Metabolomic Analyses of a Piezosensitive Mutant of Saccharomyces cerevisiae and Application for Generation of Piezosensitive Niigata-Sake Yeast Strains’, provide a screening method for generating a piezosensitive yeast mutant as well as insight into a new method of applying HHP pasteurization.
(3) Food quality and safety are important drivers in consumers preferences, and they also govern rice production and processing. Green food is a certification level that ensures sustainable development, meeting the standard operational protocols throughout the whole industry chain. Despite the growing interest in green rice, there is limited information about its quality. Xu et al. [8], in ‘Green Labelled Rice Shows a Higher Nutritional and Physiochemical Quality Than Conventional Rice in China’, showed, using the Daohuaxiang 2 variety, very interesting results regarding physical features, chemical composition and rheological properties.
(4) Physical treatments of cereals allow for modifying grains’ properties, and is a very interesting alternative to obtaining new products. Lately, pulsed electric fields offer the possibility to improve food quality and stability. Qiu et al. [9] studied the ‘Changes in the Glutinous Rice Grain and Physicochemical Properties of Its Starch upon Moderate Treatment with Pulsed Electric Field’, highlighting how the field’s strength affects the surface structure of rice grains, and also its impact on gelatinization. The authors extended the study to understand the impact of said technique on proteins and starch structure.
(5) Rapid and objective quality evaluation methods of rice based on physicochemical measurements are necessary. Nakamura et al. [10] compared the pasting properties of various rice samples using three different heating and cooling programs using a newly developed, high-temperature-type, Rapid Visco Analyzer (RVA), and investigated the relationship between the pasting properties and starch microstructure, physical properties of the cooked rice, and prolamin ratio. They developed estimation formulae for the retrograded hardness of rice based on the pasting property. This led to an easy and rapid assay method for the cooking properties of various rice samples.
(6) Rice is one of the most commonly consumed grains. It can be a good source of nutrients, but its consumption could also contribute to exposure to toxic elements. Bielecka et al. [11] evaluated 99 samples of rice and rice products in Poland for the content of As, Cd, Pb, and Hg. The values of health risk indicators did not show an increased risk to the Polish adult population. However, a daily intake of 55 g of rice corresponds to the benchmark dose lower confidence limit (BMDL) for Pb. The studied rice products can be regarded as safe for consumption by the Polish population as far as the content of As, Cd, Pb, and Hg is concerned.
(7) The epidemiology of chronic kidney disease (CKD) is showing increasing trends in prevalence and mortality, and has become a leading health problem worldwide. Reducing the proteins ingested from rice is an easy way to control the total intake of proteins in rice-eating countries. Watanabe and Ohtsubo [12] reviewed the brief history of low-protein dietary therapy for renal diseases and the recent development of low-protein processed brown rice (LPBR). This new LPBR is suggested to prevent CKD progression and enhance the quality of life (QOL) of patients with CKD.
